# The Emergence and Dynamics of Tick-Borne Encephalitis Virus in a New Endemic Region in Southern Germany

**DOI:** 10.3390/microorganisms10112125

**Published:** 2022-10-27

**Authors:** Daniel Lang, Lidia Chitimia-Dobler, Malena Bestehorn-Willmann, Alexander Lindau, Marco Drehmann, Gabriele Stroppel, Helga Hengge, Ute Mackenstedt, Klaus Kaier, Gerhard Dobler, Johannes Borde

**Affiliations:** 1 National Reference Laboratory for TBEV, Bundeswehr Institute for Microbiology, 80937 Munich, Germany; 2 Department of Parasitology, University of Hohenheim, 70599 Stuttgart, Germany; 3 Public Health Office, District Ravensburg, 88212 Ravensburg, Germany; 4 Institute of Medical Biometry and Statistics (IMBI), University Medical Center Freiburg im Breisgau, 79106 Freiburg im Breisgau, Germany; 5 Praxis Prof. Borde & Kollegen, Gesundheitszentrum Oberkirch, 77704 Oberkirch, Germany; 6 Division of Infectious Diseases, Department of Internal Medicine, University Medical Center Freiburg im Breisgau, 79106 Freiburg im Breisgau, Germany

**Keywords:** tick-borne encephalitis, emerging, TBEV, TBE

## Abstract

Tick-borne encephalitis (TBE) is the most important viral tick-borne infection in Europe and Asia. It is emerging in new areas. The mechanisms of emergence are fairly unknown or speculative. In the Ravensburg district in southern Germany, TBE emerged, mainly over the last five years. Here, we analyzed the underlying epidemiology in humans. The resulting identified natural foci of the causal TBE virus (TBEV) were genetically characterized. We sampled 13 potential infection sites at these foci and detected TBEV in ticks (*Ixodes ricinus*) at eight sites. Phylogenetic analysis spurred the introduction of at least four distinct TBEV lineages of the European subtype into the Ravensburg district over the last few years. In two instances, a continuous spread of these virus strains over up to 10 km was observed.

## 1. Introduction

Tick-borne encephalitis (TBE) is the most important tick-borne viral infection in Europe and Asia [1]. The disease is caused by a flavivirus tick-borne encephalitis virus (TBEV). There are at least three acknowledged TBEV subtypes (European, Siberian, and Far Eastern) and two or three genetically distinguishable candidate subtypes (Baikalian, Himalayan, subtype 178-19) [2,3,4,5]. The origin of TBEV and its spread in Asia and Europe have been discussed for decades. The advances in molecular biology, especially in sequencing technologies, made sequence data on TBEV partial and complete genomes available, facilitating phylogenetic and evolutionary analyses regarding the origin and spread of TBEV. One of the most comprehensive TBEV studies shows that TBEV originates from the Siberian part of Russia, spreading afterwards in several directions which form the European subtype (including Louping ill virus, LIV) and its strains (the Siberian and the Far-Eastern subtypes of TBEV) [6].

More detailed regional studies, mainly on the distribution of the Siberian subtype of TBEV, show that anthropogenic activities may play an important role in the dispersal of TBEV along man-made traffic aisles from Siberia to the Baltics [7]. According to these analyses, the Siberian subtype of TBEV originated about 300 years ago in central Siberia. However, other analyses indicate the origin of the Baltic group of the Siberian subtype of TBEV in the Baltic region itself [8]. For the European subtype of TBEV, similar phylogeographic and evolutionary analyses are rare. An analysis incorporating TBEV strains from Central Europe (Czech Republic, Slovak Republic, Germany, and Austria) demonstrated a temporo-spatial evolution of TBEV for more than ~350 years. The dissemination of TBEV in an east-western or west-eastern direction was proposed and is still a matter of debate [9,10].

The above-mentioned studies have several limitations as they are based on a retrospective design, mainly comprising historic virus strains (to some extent with unknown or non-comparable isolation/cultivation histories) and largely lack a systematic sampling of natural TBEV diversity. Analyses using extant TBEV strains, specifically isolated for phylogenetic analysis, are largely missing. Over the last few years, TBEV and human TBE diseases were recorded with increasing numbers in several countries where TBE was absent before [11,12,13,14,15]. In the historically well-known TBEV endemic areas, changes in the number of reported human TBE cases are usually used as a surrogate for the emergence or disappearance of TBE and TBEV. In Austria, where surveillance data on the dynamics of human TBE cases have been available for more than 40 years, TBEV infections disappeared in certain parts of the country and emerged in other regions [16]. A 2007 study was not able to detect the *Neudörfl* genotype (one of the TBEV-type strains) anymore in the area where it was initially isolated in 1970 [17]. In the present study, we identified a district in southern Germany with an increasing number of human TBE cases, especially in the last 5 years. TBEV natural foci of these emergent cases were identified epidemiologically based the patients’ history regarding the location of the suspected or proven tick bites. Ticks were sampled and TBEV strains were detected from the identified TBEV foci. The envelope genes (*E* genes) of TBEV were sequenced directly from TBEV-positive tick homogenates and phylogenetically characterized, aiming at a better understanding of the introduction mode and dissemination in an emerging TBEV endemic site.

## 2. Material and Methods

### 2.1. Case Definition and Case Data

Since 2001, TBEV infections in humans have constituted a notifiable disease in Germany. Detailed case definitions—based on serological testing—are issued by the Robert-Koch Institute (RKI) and European Centre for Disease Prevention and Control (ECDC). It must be taken into consideration that the German case definition also includes all cases of laboratory-confirmed TBEV infection, independent of the clinical course. Case numbers are provided by the RKI via an online search tool, SurvStat (SurvStat 2.0), which offers open access.

### 2.2. Statistical Analysis

Case numbers were retrieved from the RKI SurvStat tool and exported to IBM SPSS (Version 28.0.0.0). All statistical calculations were carried out by using IBM SPSS, descriptive statistics, paired *t*-tests, and unpaired *t*-tests. Forecasting was applied using linear time series methods with the application of Brown’s simple exponential smoothing model. Student’s *t*-test was used to compare datasets, *p* is defined: * *p* < 0.05; ** *p* < 0.01; *** *p* < 0.001. The upper and lower limits of 95% CIs were displayed.

### 2.3. Identification of TBEV Natural Foci

**Figure 1:**
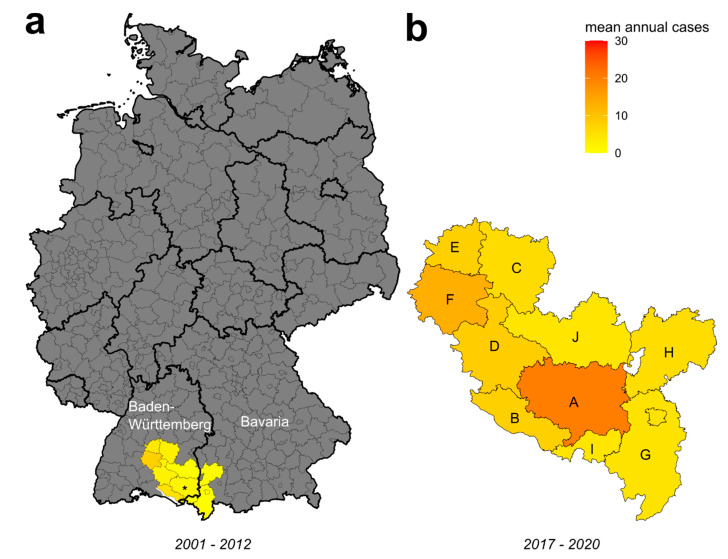
Recent rise in mean annual TBEV cases in Southern Germany. (**a**) Federal map of Germany displaying the location and mean annual reported case numbers for Ravensburg and its nine neighboring districts in the federal states of Baden-Württemberg and Bavaria from 2001 to 2012. An asterisk indicates the location of the Ravensburg district. The color scale encodes the mean annual case numbers. The missing values are depicted in gray. The black and light gray contours depict state and district boundaries, respectively. (**b**) Enlarged map of the ten monitored southern districts displaying the mean annual number of reported TBEV cases from 2017 to 2020. Depicted districts are listed in Table 1 (district ID; A–J). The color scale is shared with (**a**).

The Ravensburg district (Figure 1b; A) is located in the south-eastern part of the German federal state of Baden-Wuerttemberg (Figure 1a), bordering on the Bavarian districts of Oberallgäu (Figure 1b; G), Unterallgäu (H), and Lindau (I) in the east; the Bodensee district in the south; and the Biberach (J) and Sigmaringen (D) districts in the north and west.

Patients with confirmed TBEV infection were contacted by the local public health authorities in the district of Ravensburg in the south-eastern part of the federal state of Baden-Württemberg. During the mandatory infection tracing process and interview, the patients were asked if they could remember the location of the tick bite within the putative incubation period. If the patient could localize the location of the suspected tick bite, the candidate sites were clarified on Google Maps and tick sampling was conducted.

### 2.4. Sampling and Processing of Ticks

Ticks were sampled by flagging and were transported alive in 50 mL plastic tubes to the laboratory. Ticks were identified morphologically, sorted according to their stage and gender, and pooled with five adults or ten nymphs per pool containing 1 mL of tick crushing medium (MEM plus 10-fold antibiotics and antimycotics plus non-essential amino acids plus 3% fetal calf serum; all cell culture reagents from Invitrogen, Karlsruhe, Germany). Ticks were kept at −80 °C until further processing. Ticks were homogenized using a Fast Prep Savant FP120 tissue lyser (Bio101, Vista, CA, USA) by three rounds at speed 6.5 for 30 s each. Between each round the tubes were cooled on ice for 3 min.

The viral RNA was extracted from the tick homogenates using the MagNA Pure LC RNA/DNA Extraction Kit in a MagNA Pure LC instrument (both from Roche, Mannheim, Germany) according to the manufacturer’s instructions. Then, 200 µL of the respective tick homogenate suspensions was extracted in a total end volume of 50 µL elution buffer. Extracted viral RNA was used at once for PCR and stored at −80 °C for further testing by PCR.

### 2.5. Sequencing and Analysis of TBEV Genomes

The *E* gene was amplified from RT-qPCR positive samples and processed for sequencing according to published protocols [18,19]. The purified *E* gene amplicons were sent to Eurofins for sequencing. The obtained sequence data were assembled using Geneious Prime^®^ 2021.1.1, generating final *E* gene sequences of 1488 bp. To place the identified TBEV isolates in the larger context of the European subtypes, for phylogenetic inference, we included 25 *E* genes of representative, historical, and geographically close isolates obtained from the NCBI Genbank database (Supplementary Table S1). Recombination detection using the full-length multiple sequence alignment was carried out with the GARD web server (http://www.datamonkey.org/GARD (accessed on 17 May 2022)) using default parameters [20,21]. For the full-length *E* genes, as well as the two predicted putative recombination tract regions, multiple sequence alignment, phylogenetic model selection, and the inference of the maximum likelihood tree topologies with 1000 bootstrap replicates using the optimal evolutionary models (full sequence: TN93 + G, AIC~8878; region 1: TN93 + G; AIC~7323; region 2: K2 + G, AIC~1577) were calculated using the software implemented in the MEGA X package [22]. In total, 31 sequences were used to generate the phylogenetic tree and all 1488 positions were used for analysis. Divergence times were estimated based on the tree topology rooted with KJ922516 and MSA of the region 1 sequence range using the TreeTime software (parameters: --gtr infer --reconstruct-tip-states --clock-filter 2 --coalescent skyline --clock-std-dev 1e-200) [23].

## 3. Results

The Zollernalbkreis (F), Reutlingen (C), Tübingen (E), and Bodensee (B) districts are adjacent to these neighboring districts in the north and west. The Ravensburg district has reported a striking increase in reported human TBE cases since 2017 (Figure 1a vs. Figure 1b). While the average number of human TBE cases was 1.6 from 2001 (the start of the national surveillance program for TBEV infections in Germany) to 2012 (19 cases in 12 years), the annual numbers increased from 2017 to more than 20 (81 cases in 4 years). A similar trend was detectable in the two neighboring districts, Lindau (15-fold increase) and Oberallgäu (15.8-fold increase; see Table 1 for descriptive statistics). However, compared to Ravensburg, the total and mean annual case numbers in both districts were substantially lower during both intervals (e.g., both 21% of total cases in Ravensburg during 2001–2012; 4 vs. 19 cases; Table 1).

**Figure 2:**
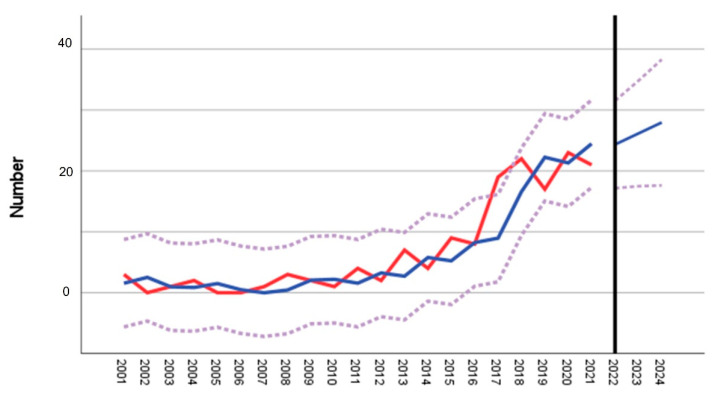
Number of notified human TBE cases spanning the 2001–2021 time period in the district of particular interest: Ravensburg forecasting the years 2022–2024. The red line indicates the notified number of cases, while the blue line indicates the forecasting curve (R^2^ = 0.819). The upper and lower limits of 95% confidence intervals are shown as dotted lines.

A time series/forecast model for Ravensburg was generated using linear time series methods by applying Brown’s simple exponential smoothing model, forecasting the years 2022–2024 (Figure 2). Without preventive interventions, the model forecasted a further increase in human cases in the coming years, i.e., suggesting almost 30 cases for the year 2024.

**Figure 3:**
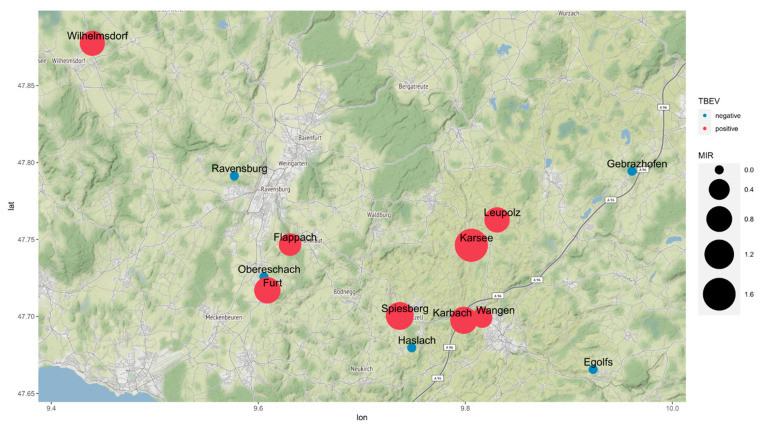
Geographical distribution of TBEV-positive *Ixodes ricinus* ticks in the Ravensburg district. Topological map of the Ravensburg area displaying a detailed localization of sampled sites. Point sizes scaled by the minimal infection rates of individual sites. The point color indicates results of the TBEV-RT-qPCR.

Ticks were collected from 2017 to 2020 at 13 different putative infection locations. In 8/13 locations, TBEV could be detected in ticks, and the TBEV *E* genes were sequenced and phylogenetically analyzed. A total of 2955 ticks were sampled, with 366 males, 292 females, and 2297 nymphs. All ticks were identified based on morphological features as *Ixodes ricinus*. Detailed information on geographical and developmental distribution of sampled ticks is provided in Figure 3 and Table 2.

Fourteen tick pools tested positive for TBEV by RT-qPCR. By postulating one TBEV- positive tick per pool, a minimal infection rate (MIR) of all sampled ticks was calculated at 0.47%. We observed significant differences in MIRs during tick development (χ^2^ test *p*-value << 0.01). While adult ticks had an overall MIR of 1.2%, females had an overall MIR of 1.8% and nymphs displayed a rate of 0.17%. After omitting the ticks of the five TBEV-negative locations, the MIRs of the confirmed natural TBEV foci increased to an overall MIR of 0.8% (2.0% males, 2.6% females, and 0.3% nymphs). In five out of eight positive natural foci, only one TBEV-positive tick pool was found, while two tick pools in one focus (Spiesberg), three tick pools in one focus (Wilhelmsdorf), and four tick pools in one focus (Leupolz) were TBEV-PCR-positive (Table 2). Overall, MIRs varied substantially between the respective natural TBEV foci, ranging from the lowest with 0.37% at Karsee to the highest with 1.7% at Wangen (Figure 3).

Full-length TBEV *E* genes could be isolated directly from tick homogenates in thirteen of the fourteen pools. Some of the microfoci displayed isolates with identical sequences. Two of the four *E* genes from Leupolz and the two genes from Spiesberg were identical in sequence and thus were each represented as a single taxon in the phylogenetic analysis.

**Figure 4:**
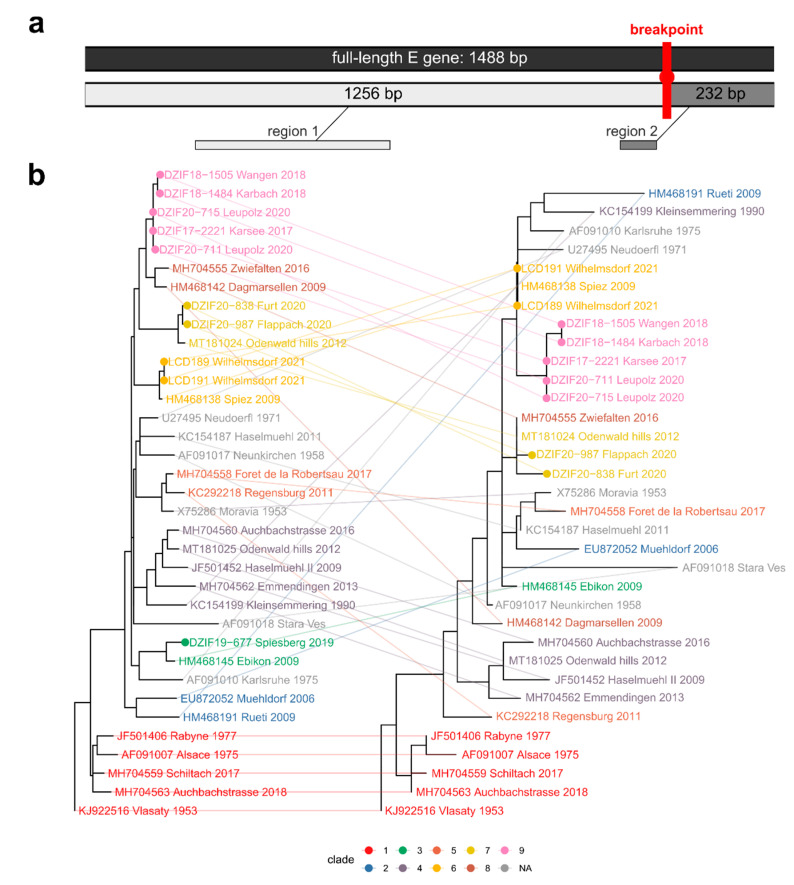
Phylogenetic inference of virus strains from the nine natural TBEV foci in the Ravensburg district in the context of representative clades from the published European TBEV *E* gene diversity. (**a**) Schematic depiction of the full *E* gene (upper gray bar) and two inferred putative recombination tracts (regions 1 and 2; lower gray bars). A red vertical line indicates the position of the predicted recombination breakpoint (at amino acid residue 699 of the TBEV polyprotein). (**b**) Co-phylogram plot of the maximum likelihood tree topologies inferred using the respective optimal model for the alignments of the two putative recombination tracts in the *E* gene as depicted in panel (**a**). Corresponding tips in the three tree topologies are displayed as clade-color-coded inter-tree edges. The ten sequenced TBEV isolates are highlighted by colored dots. Significantly supported phylogenetic clades (bootstrap support > 70%) in the phylogenetic analysis of sequence region 1 are indicated via coloring of tip labels and inter-tree connections as depicted in the bottom legend. Gray coloring (NA) indicates singleton taxa, which did not cluster in such a significant clade. The topology of the tree inferred from the full-length alignment (not shown) is almost identical to that of region 1.

The phylogenetic trees derived from full-length multiple sequence alignment (MSA) of the *E* gene displayed lower phylogenetic resolution in the backbone and specific clades (Supplementary Figure S1). To eliminate possible confounding signals, we searched for potential recombination events in the MSA of 36 *E* genes comprising the ten strains from the Ravensburg district as well as representative members of the sampled TBEV *E* gene diversity from the NCBI Genbank database. The genetic algorithm implemented in GARD suggested one recombination breakpoint at position 1256 of the *E* gene (Figure 4a) [20]. The potential breakpoint resides in the stem region of the E glycoprotein’s transmembrane (TM) anchor or TM hairpin (NCBI CDD database entry: flavi_E_stem) which is important for membrane fusion [24]. Region *2* contains part of the stem region, as well as the complete remainder of the hairpin structure spanning two TM domains and an extracellular loop. Region 1 comprises the cytoplasmatic parts of the E glycoprotein with the conserved central and dimerization domains (*Flavi_glycoprot*) harboring the immunoglobulin-like domain III (*Flavi_E_C*) which is important for interaction within the host–cell surface [25].

**Figure 5:**
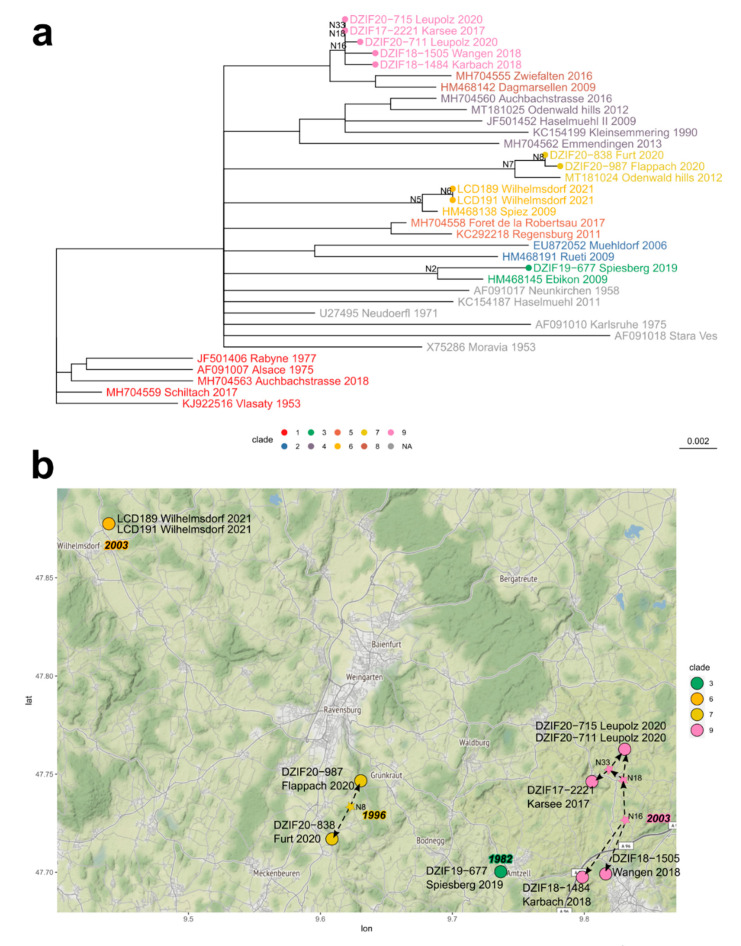
Phylogeography of virus strains from the eight natural TBEV foci in the Ravensburg district. (**a**) Chronogram of the maximum likelihood tree topology of the putative *E* gene recombination region 1 (see also Figure 1a). Rooted using KJ922516.1 (Vlasaty, CZ, 1953, in clade 1). Divergence times estimated using TreeTime. Internal tree nodes with a bootstrap support below 70% were collapsed. Tip labels are color-coded by evolutionary clade (see color legend). Divergence time estimates of the annotated internal nodes (e.g., N2) are presented in Table 3. The sampled taxa from the eight natural foci of the Ravensburg area are highlighted by node tip points colored by clade. (**b**) Geographical map of the Ravensburg district in Germany. Sampled taxa of the focus areas are color-coded by evolutionary clade. Focus area 1/clade 9: Wangen/Karsee/Karbach/Leupolz; focus area 2/clade 7: Flappach/Furt; focus area 3/clade 6: Wilhelmsdorf; focus area 4/clade 3: Spiesberg. Small stars that connect to the tip points of the sampled isolates via dashed arrow lines and are filled by the same clade color depict putative ancestral states for the two microfoci with multiple isolates. These data points do not represent geographical location but display the microfocus divergence patterns showing the internal dated nodes of the clade in the tree shown in panel 4a. Years given in the color-coded bold font corresponding to each focus represent the estimated divergence dates of the respective clade’s oldest internal node (e.g., clade 7/focus 2: node N7: 1996). Thus, these dates represent the maximum age of the clade and upper boundary for the emergence of the respective TBEV focus area.

To account for possible impact on the phylogenetic signal in those regions, we conducted the subsequent phylogenetic analyses on the individual MSAs of the full-length *E* gene sequence as well as the sequence regions of the two putative recombination tracts separately. The phylogenetic analyses comprised a selection of the respective optimal nucleotide substitution model and the subsequent phylogenetic inference of the maximum likelihood tree topology for each of the three MSAs. We assessed the robustness of tip clustering based on bootstrap support values (>70%) to define significant phylogenetic clades representing genotypic lineages (expressed by specific tip colors in Figure 4b and Figure 5).

Comparing the composition of significant clades in the three trees, we observed an overall large consistency between the full-length and region 1 tree topologies (Figure 4b). While the topology of region 2 for the genotypes isolated from the Ravensburg focus areas remained largely unaltered, it differed for some of the reference strains from the NCBI database. Comparing to previously published E gene topologies as well as those inferred for TBEV full-genomes, we found the region 1 topology to be the most robust representation of the TBEV species phylogeny and focused on it for the subsequent analyses of our dataset [4,26]. Considering this comparison to existing phylogenetic analyses and the samples’ reported collection dates, we rooted the region 1 tree using the Central Bohemian (CZ) Vlasaty 1953 genotype and estimated divergence times (Figure 5a and Table 3) [27].

The combined phylogenetic evidence suggests that the TBEV strains detected in ticks in the Ravensburg district originated from four distinct independent sources, corresponding to four significantly supported phylogenetic clades. The TBEV isolates from the microfoci Karsee, Wangen, Karbach, and Leupolz represent a clade of common descent that was predicted to have emerged sometime between 1989 and 2008 (point estimate: 2003). These genetically closely related TBEV strains are isolated from foci, about 6 km apart, thus forming a TBEV focus area which, in ecological or population genetic terms, corresponds to a deme with a TBEV genotype of common descent (Figure 4b, *focus area 1*; *clade 9*). While the two more southern isolates from ticks in Karbach and Wangen may be derived from the initial introduction into the area around 2003, the phylogeographic and dynamic analyses imply a common ancestor of the Karsee/Leupolz isolates within the area sometime between 2003 and 2017, possibly in 2006. Given that the last common ancestor (LCA) with direct sister clade comprising strains from the 60 km distant alimentary outbreak in Zwiefalten (2016; [21]) and a 2009 Swiss strain from Dagmarsellen [28] was dated to have emerged back in 1962, we can exclude a recent direct connection to these cases.

A second clade is formed by the 2020 TBEV strains Furt and Flappach that cluster robustly with a 2012 strain from the Odenwald hills (clade 7, Figure 5a) in the northern part of the state in the border regions of Baden-Württemberg, Bavaria, and Hessen. The two microfoci of Flappach and Furt are about 3 km apart and thus form a distinct natural TBEV focus area with a genotype of common descent (Figure 4; Table 3; focus area 2). While the emergence of the LCA (node *N7* in Table 3) dates back to 1996, the LCA of the focus area is predicted to have emerged more recently (2014; node N8 in Figure 5). The predicted substantially older timing of the LCA and distance to the Odenwald speaks against a direct connection of the cases and instead suggests a common yet-unknown source, potentially localized in one of the intermediate districts.

The third clade comprises the 2021 TBEV strains of Wilhelmsdorf (Figure 4 and Table 3, focus area 3, clade 6) that cluster robustly with a strain isolated 2009 in Spiez in the Canton Bern in central Switzerland [29]. The LCA of the clade dates to 2003 and the virus might have been introduced into the focus area already in 2017. The cluster is genetically, topologically, and geographically clearly disjunct from other focus areas (16 km to Furt /Flappach; 32 km to Wangen/Karsee/Karbach/Leupolz) and thus forms an additional natural TBEV focus area. This is the most western TBEV focus of the district. So far, this TBEV virus clade has only been detected once in the area, and it is therefore unclear whether it has a larger distribution beyond Wilhelmsdorf. However, the timing of the LCAs (nodes N6 and N5 in Figure 5 and Table 3) suggests an earlier introduction into the focus area, possibly from Switzerland.

The fourth distinct clade observed in the Ravensburg district includes the sequence from the natural focus Spiesberg (Figure 4, focus area 4). The *E* gene sequence of this TBEV genotype clusters with a different Swiss strain that was isolated in Ebikon in 2009 [29,30]. Since the LCA of the clade dates back to 1982, we can exclude a direct connection between the cases. Although the focus area is geographically in close proximity to the TBEV natural focus area 1 (8 km to Wangen/Karbach; 8 km to Karsee/Leupolz), it is genetically distinct and very likely sourced independently, and there is no evidence for gene flow between the focus areas. We cannot rule out an introduction from Switzerland, but given the timing of the LCA and the sampling date of the Ebikon isolate, a more direct connection between the two foci appears highly unlikely.

Strikingly, none of the other TBEV strains which were isolated over the last few years in south-western Germany (Emmendingen, Aubachstrasse) and neighboring France (Alsace) seem to be closely related to the strains in the district [26,30,31,32].

**Figure 6:**
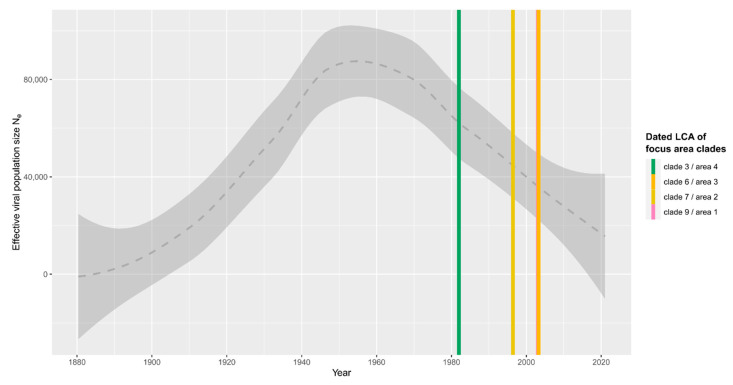
Coalescent Bayesian skyline plot modelling the TBEV population dynamics based on the chronogram of the E gene region 1 shown in Figure 5. The skyline (gray dashed line) was modelled with TreeTime, assuming 50 gen/year and approximate confidence bounds (±2 standard deviations; gray band). Colored vertical lines mark the estimated divergence times of the independent LCAs of the four TBEV clades/focus areas.

We estimated TBEV population dynamics based on the dated maximum likelihood tree topology of the E gene region 1 using the coalescent modelling approach implemented in TreeTime. The resulting Bayesian skyline plot depicting the estimated effective population size (N_e_) of TBEV over time is shown in Figure 6.

## 4. Discussion

TBE is an emerging virus infection in many parts of Europe. The exact mechanisms for the emergence of TBEV, and consequently human TBE in new geographic locations, are largely unknown. For some TBEV strains, it could be shown that human activities might be at least partially important [7,8]. A phylogenetic analysis on the recent introduction of TBEV on the British Isles implied an introduction by birds [15]. Similar importations of TBEV by migrating birds are suggested by phylogenetic studies of TBEV, e.g., in Finland and Japan [33,34]. The establishment of new permanent foci may depend on complex environmental requirements [35]. Among them, climate and climate change have been mentioned as important factors for the expansion or emergence of new TBEV foci [36,37].

Changes in land cover and land use are discussed as potentially major factors for the emergence of vector-borne diseases, especially tick-borne diseases [38].

New TBEV foci are usually defined based on the appearance and dynamics of human TBE cases. Besides the new introduction of TBEV, the activation of an existing TBE focus might also cause an abrupt increase in human cases. Humans have to encounter and invade a TBEV focus to become infected, and variations in human sociological behavior may cause a sudden (re-)emergence or increase in TBE cases due to an existing TBEV natural focus which is newly invaded or (re-)invaded [39,40].

In Ravensburg, some of the above-mentioned reasons may be excluded. There was no obvious change in land cover or land use during the very short time period of only about five years, with particularly increasing case numbers. There are no indications for a significant shift in social behavior in this rurally structured area. The changes in human behavior during the COVID-19 pandemic, accompanied by a dramatic increase and subsequent normalization in the frequentation of local natural recreation areas during and after the lockdown phases, provide an ideal platform to assess the impact of this factor. In the year 2020, when the overall human TBE cases in Germany peaked during the lockdowns, the number of human TBE cases did not similarly increase in the Ravensburg district (RKI, SurvSTAT). In 2021, a remarkable drop in human TBE cases was recorded in Germany, especially in the federal state of Baden-Wuerttemberg (2020: 336 cases, 2021: 144 cases). This significant decrease in human TBE cases was not observed in the district of Ravensburg, where there was only a decrease from 23 to 21 human cases. These data argue against a major anthropogenic factor in the introduction or spread of TBEV and we speculate that the TBEV situation in the Ravensburg district, in comparison to the neighboring districts, is somehow unique and the responsible factors are unclear at the moment.

Regarding environmental variables, there is no indication that meteorological factors are major drivers of the specific situation in the district. The years 2018 to 2020 were the three warmest years in Germany since the start of the weather data recordings, with average temperatures of >10 °C in all three years (DWD, German Weather Service). The rising trend of human TBE cases in Ravensburg, however, already started before 2018. In 2021, the average temperature was again at 9.5 °C, reaching a normal range as before the three hot years from 2018 to 2020. Nevertheless, in that year, the Ravensburg district registered the second highest case number ever. We cannot exclude that the observed increase may be due to the delayed effects of warm years from 2018 to 2020. Previous work from Brugger et al. investigated weather influences on TBEV and on the biology of the vector in different models. Statistics found a higher correlation of tick densities with time-lagged and temporal variables than with contemporaneous explanatory variables [41]. The development of recorded human TBE cases in the coming years in Ravensburg is under particular surveillance. As plotted in our time series forecast (Figure 1) from a statistical perspective, a further increase has to be anticipated. Although the selection and number of TBEV natural foci are not representative, there is no evidence that altitude as a surrogate for warmer climate may play a role in Ravensburg at this stage in our field investigations [42,43]. However, this development is a matter for future research over the next few years, even if more new TBE natural foci will be identified.

Despite the absence of obvious environmental factors, the recent rising emergence of TBE cases in the Ravensburg district is striking and yet might be only explainable by a closer investigation of additional secondary environmental factors. A similar development is detectable at a lower level in the neighboring districts. The Lindau district (south and west of Ravensburg) has a comparably high increase in human TBE cases. Unfortunately, no TBEV strains are yet available from this district. In addition, the district of Oberallgäu in the east of Ravensburg exhibits a high increase in human cases. Again, so far, neither the natural foci nor any TBEV strains can be identified. The geography of these districts at the northern edge of the Alps may allude to some environmental factors associated, e.g., with the topology or climate at the northern rim of the mountains. A similar observation can be made in Austria, where TBEV, measured in human TBE cases, migrated from eastern federal states to the western Austrian federal states of Salzburg, Tirol, and Vorarlberg. Geographically, Vorarlberg is in close proximity to the Ravensburg foci with increasing TBE activity in the south [16]. Another example coupling such a geographic scenario with increasing human TBE cases is seen on the northern rim of the low mountain range of Erzgebirge in central Germany. There, human cases have increased over the past few years. Thus, we can assume that yet-unidentified environmental factors, both biotic and/or abiotic, impact the establishment and persistence of novel TBEV foci, potentially establishing stable natural cycles.

The current albeit early stage of TBEV surveillance in the area suggests that these foci appear to be highly restricted to certain specific localities, and with no evidence for gene flow beyond 6–10 km (Figure 5). The identified four natural TBEV foci represent four distinct phylogenetic clades of TBEV with recent common independent ancestors that have likely diverged at disjunct times during the course of TBEV evolution in Europe.

The analysis of TBEV population dynamics, given the inferred chronogram of the sampled strains (Figure 6), indicates an expansion and a peak of effective population size (N_e_) up to the 1970s, followed by a subsequent decline in TBEV N_e_ that continues into the present day. This estimated decline in N_e_ is highly consistent with our observation of highly localized natural TBEV foci formed by genetically distinct gene pools. Presumably, these dynamics might reflect altered population and migration dynamics of the two major TBEV hosts, i.e., rodents and ticks. Since the 1970s, at the latest, the natural boundaries between distinct populations of these hosts have certainly been increasingly affected by the anthropological impact on the environment. Consequentially, long-range TBEV dispersal may increasingly depend on random-chance events where infected ticks feed on off-target hosts with larger mobility ranges, e.g., migratory birds, deer, or even humans, resulting in the observed a low N_e_.

In line with these observations, there are no high MIRs (i.e., low absolute population sizes) in our sample pools, which contradicts the hypothesis of a higher or increasing activity of TBEV in ticks. The observed MIRs for the Ravensburg foci are only slightly higher than documented by previous surveillance studies [44,45,46]. Furthermore, in these studies, ticks were randomly sampled and not investigated at defined potential TBEV natural foci. The presented findings confirm the struggle to detect TBEV at potential sites surrounding human TBE cases due to the very low prevalence rates of TBEV in ticks. Compared to other reports on MIRs in defined TBEV foci, our MIR numbers are within the ranges observed in other Central European TBEV natural foci [19,26,31,47].

From a population genetics perspective, the observation of small population sizes raises questions around whether these disjunct TBEV populations are bottlenecked and, if so, how the integrity of the RNA genome is maintained despite the relatively high mutation rates.

Given the taxon sampling of European and particularly German TBEV genotypes that are still geographically restricted, the presented phylogenetic analysis of the different TBEV strains allows only limited speculation about the origins of the Ravensburg TBEV foci and about their introduction into the district. Considering these restrictions, we hypothesize a slow but continuous local spread along the landscape structures (river valley, forest edge) that may be limited by natural boundaries of the two major TBEV host species.

The results from molecular phylogenetics, dating, and phylogeography speak in favor of the view that TBEV long-distance dispersal might be dependent on less frequent random-chance encounters of infected ticks feeding on hosts with larger migratory ranges.

For example, for the TBEV clade 9 genotypes found in focus area 1 (Wangen/Karbach/Karsee/Leupolz), molecular divergence time estimates place the last common ancestor with the sister clade comprised of more remote genotypes from Zwiefalten (~80 km) and Dagmarsellen (~180 km) to the 1960s. Thus, placement around the presumed peak expansion period of European TBEV diversity (Figure 6) is possibly connected to the early expansion of TBEV occurrences in the Czech Republic. The divergence dating speaks against a more recent direct connection of the cases, as we would expect in an instance of long-range TBEV dispersal.

Our data also provide initial insight into the dynamics and timing of the local spread of a natural TBEV focus. According to our estimates, the LCA of focus area 1 dates back fourteen years prior to the first collected genotype in 2017. Thus, the genotype could already have been slowly spreading in the area since 2003. The current focus area ranges over 6 km from north to south along the valley of the small river Karbach. According to the registration of the District Public Health Office, some more human TBE cases have been registered over the last few years along the river Untere Argen, which runs through the city of Wangen, into which the river Karbach flows west of the city of Wangen. We hypothesize that the continuous spread of the TBEV genotype along the little creek valley could represent a possible route of gradual dispersal, reflecting the limited movement of small mammals. Fragmented forests and forest bands, including these little valleys, may facilitate the dispersal of the TBEV strain, as was also shown for other TBE foci in southeastern Germany (publication in preparation). This dispersal of the genotype to different locations, as well as the establishment and concentration of new TBE foci in a limited focus area, might be the main reason for the emergence of human TBE over the last few years. Similar concentrations of TBE foci are also observed in the highly endemic districts of eastern Bavaria and Czech Republic [9,10], although their importance for hyperendemic TBE areas only now becomes more obvious.

From a methodological perspective, our analyses highlighted an important aspect for the use of the *E* gene as a phylogenetic marker to trace TBEV evolution or population dynamics. Given the low bootstrap support, especially towards the backbone of the tree that seems to be a common phenomenon in TBEV phylogenetics [48], we utilized a recombination breakpoint analysis as an analytic tool to identify regions with conflicting evolutionary signals. The existence and importance of recombination in shaping TBEV diversity is a controversial topic [48,49]. Acknowledging this fact in light of the reproducibility for the journal audience, as well as the scope and focused aim of this study, we refrained from a more detailed elucidation and validation of the predicted putative recombination breakpoint in the TBEV *E* gene region. In our view, such an endeavor would require a larger taxon set and ideally compare whole genomic sequences, clearly going beyond the scope of this study that focused on the population dynamics and divergence patterns concerning the emergence of the described focus areas in a novel German TBEV hotspot. When we limit our analysis to the alignment of the first predicted putative recombination tract (region 1), the bootstrap support and topology significantly improved compared to the full-length, demonstrating the utility to restrict the phylogeny on this part of the *E* gene.

In our interpretation, the identified breakpoint may likely not be the result of a true recombination event, but rather may represent a drop or change in the phylogenetic signal due to relaxed or altered selective pressure that may act differently on the stem region and hairpin structure of the *E* gene’s TM domains. Such an altered signal might be also be important to consider in other population-level analyses relying on the *E* gene marker or even in other taxa.

## 5. Conclusions

The district of Ravensburg evolved within the last 5 years to one of the districts with the highest incidence of TBE in Germany. The detection of eight TBE foci and the phylogenetic analysis revealed information about the origin and introduction of the new local TBEV situation. TBEV was likely introduced at least four times independently into the district, establishing TBEV foci that form four distinct TBEV focus areas. In two of these areas, the TBEV genotypes seem to be dispersed locally along landscape characteristics such as river valleys and fragmented forest structures. The concentration of newly established TBEV foci might be responsible for the highly endemic situation in the district. Other factors such as changing human behavior, climate change, fluctuating tick activity, or virus prevalence in the wild do not appear to be primary cause of this highly endemic situation. Based on the epidemiological data and the developed model, a further increase in human TBE cases in the coming three years can be forecast, probably due to the continuing dispersal of TBEV in the landscape and a formation of further TBE foci in the intensively cultivated agricultural and forested fragmented area. The detailed environmental variables facilitating this spread of TBEV strains remain to be elucidated. However, the district of Ravensburg seems to be an ideal field exploratory area for studying the introduction and spread of TBEV in different natural settings and environmental conditions.

## Figures and Tables

**Table 1:** Numbers of reported total and average annual human TBE cases and fold increases in the Ravensburg district and neighboring districts—time periods of 2001–2012 and 2017–2020 (according to the data of SurvSTAT, RKI, Berlin, Germany) (^+^ in 2017 alimentary/milk outbreak with 14 notified human TBE cases; numbers in brackets calculated without alimentary cases). Student’s *t*-test was applied to compare the two time periods. * *p* < 0.05; ** *p* < 0.01; *** *p* < 0.001. R^2^ is shown for every time series/forecasting model, using case numbers spanning the 2001–2021 period.

District ID (Figure 1b)	District	Cases 2001–2012	Mean 2001–2012	Cases 2017–2020	Mean 2017–2020	Fold Increase	*t*-Test p	Time Series/Forecast Model 2001–2021 R^2^
A	Ravensburg	19	1.6	81	20.3	13.0	<0.001 ***	0.819
B	Bodensee	60	5.0	31	7.8	1.6	0.027 *	0.79
C	Reutlingen	30	2.5	24	6	2.4	0.009 **	0.034
D	Sigmaringen	25	2.1	33	8.3	3.9	0.02 *	0.102
E	Tübingen	48	4.0	31 (17) ^+^	7.8 (4.3) ^+^	0.5 (0.9) ^+^	n.d.	n.d.
F	Zollernalb	81	7.8	53	13.3	1.7	0.011 *	0.027
G	Oberallgäu	4	0.3	19	4.75	15.8	<0.001 ***	0.321
H	Unterallgäu	11	0.9	23	5.8	6.4	0.071	−0.014
I	Lindau	4	0.3	18	4.5	15	<0.001 ***	0.589
J	Biberach	7	0.6	18	4.5	7.7	0.027 *	0.103

**Table 2:** Number of sampled ticks, tabulated by developmental stage and location. Positive locations are in bold font. Numbers in brackets indicate the number of TBEV-positive tick pools.

Location	Altitude (m above s.l.)	Sampling Date	Males	Females	Nymphs	Total MIR (%)
**Karsee**	**635**	**26 September 2017**	**32 (1)**	**20**	**8**	**1.7**
**Wangen**	**573**	**27 June 2018**	**24 (1)**	**14**	**233**	**0.37**
**Karbach**	**579**	**27 June 2018**	**14**	**15 (1)**	**77**	**0.94**
Ravensburg	510	16 July 2018	8	7	150	0
Gebrazhofen	679	16 July 2018	26	19	169	0
**Spiesberg**	**539**	**03 July 2019**	**10**	**19 (1)**	**168 (1)**	**1.0**
Haslach	667	03 July 2019	27	23	285	0
Egolfs	646	28 May 2020	51	47	229	0
**Leupolz**	**601**	**23 July 2020**	**100 (2)**	**79 (2)**	**346**	**0.76**
Obereschach	485	19 August 2020	2	5	39	0
**Furt**	**496**	**19 August 2020**	**6**	**0**	**110 (1)**	**0.86**
**Flappach**	**556**	**14 September 2020**	**35**	**30 (1)**	**127**	**0.52**
**Wilhelmsdorf**	**679**	**2018/2019**	**31 (1)**	**14**	**356 (2)**	**0.75**
**Total**			**366 (5)**	**292 (5)**	**2297 (4)**	**0.47**

**Table 3:** Inferred divergence dates for the relevant internal nodes and tips of the Ravensburg focus areas. Node names correspond to annotations depicted in Figure 5a.

Focus Area			Year	90% Confidence Interval		
	Clade	Node/Taxon	Estimate	Min	Max	Location
1	9	N16	2003	1989	2008	
		DZIF18-1484	2018	2018	2018	Karbach
		DZIF18-1505	2018	2018	2018	Wangen
		N18	2006	1999	2015	
		DZIF20-711	2020	2020	2020	Leupolz
		N33	2014	2007	2017	
		DZIF17-2221	2017	2017	2017	Karsee
		DZIF20-715	2020	2020	2020	Leupolz
2	7	N7	1996	1983	2006	
		MT181024	2012	2012	2012	Odenwald
		N8	2014	2004	2018	
		DZIF20-987	2020	2020	2020	Flappach
		DZIF20-838	2020	2020	2020	Furt
3	6	N5	2003	1987	2007	
		HM468138	2009	2009	2009	Spiez
		N6	2017	2006	2020	
		LCD191	2021	2021	2021	Wilhelmsdorf
		LCD189	2021	2021	2021	Wilhelmsdorf
4	3	N2	1982	1966	1998	
		HM468145	2009	2009	2009	Ebikon
		DZIF19-677	2019	2019	2019	Spiesberg

## Data Availability

Nucleic acid sequences will be made available on the NCBI nucleotide database. Geographic data of TBE foci will be made available by the authors on request.

## Supplementary Materials

The following are available online at https://www.mdpi.com/article/10.3390/microorganisms10112125/s1, Supplementary Table S1: xlsx spreadsheet with metadata of the genotypes used in the presented phylogenetic analyses. Supplementary Figure S1: Extended version of Figure 4 includes (**b**) the tree topology based on the phylogenetic analysis of the full-length MSA (using the same methods as for (**a**) regions 1 and 2; applied optimal model: TN93 + G, AIC~8878).
