# Supplementary material for: The Emergence and Dynamics of Tick-Borne Encephalitis Virus in a New Endemic Region in Southern Germany
Source: Microorganisms. 2022 Oct 27;10(11):2125. doi: 10.3390/microorganisms10112125 (PMC9693875; doi:10.3390/microorganisms10112125)
Supplement: Supplementary file 1 [file microorganisms-10-02125-s001.zip › microorganisms-1892497-suppl. Figure S1.pdf]

microorganisms

MDPI

*Supplementary materials*

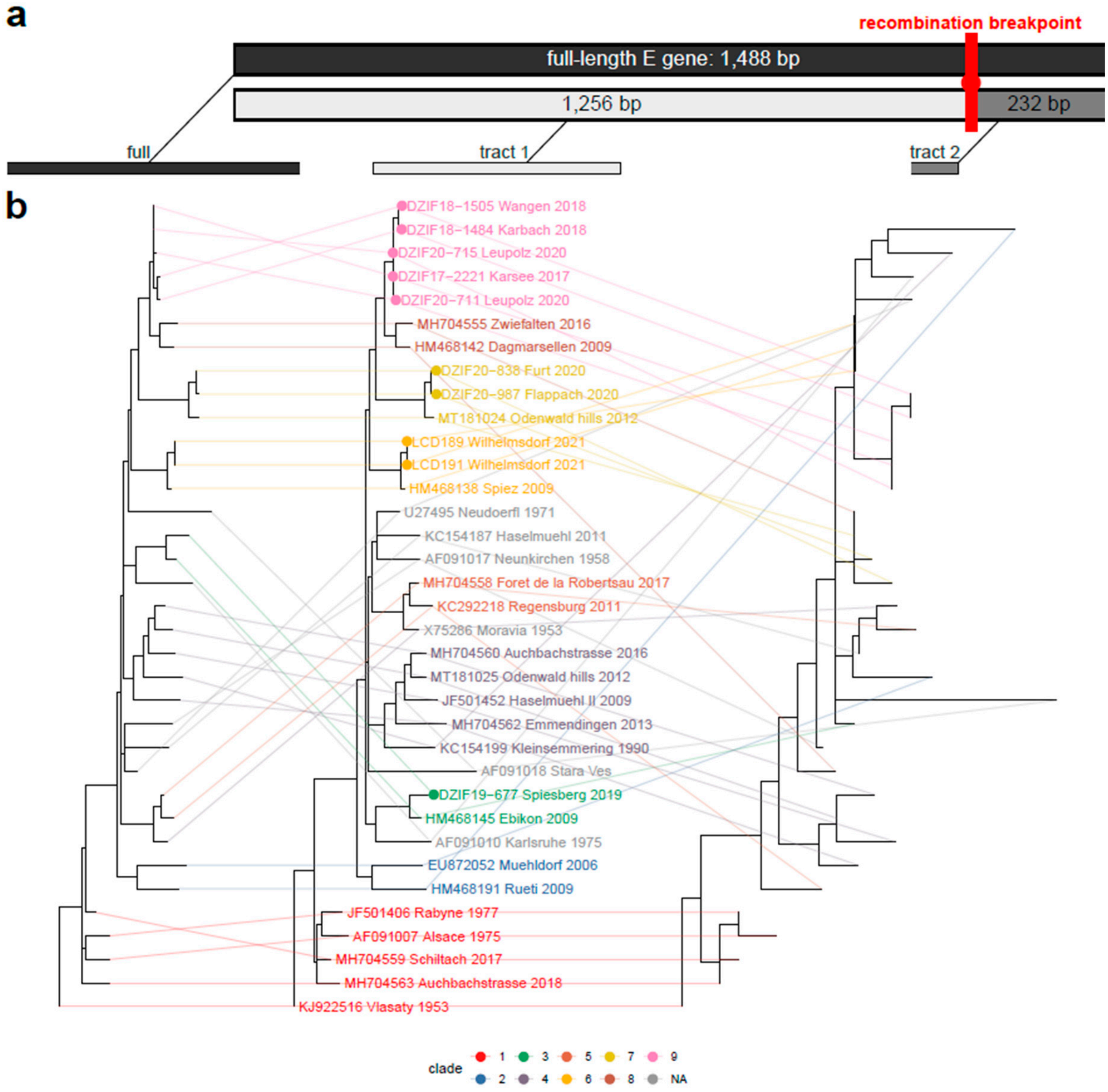


**Figure S1.** Extended version of Figure 4 includes (**b**) the tree topology based on the phylogenetic analysis of the full-length MSA (using the same methods as for (**a**) regions 1 and 2; applied optimal model: TN93 + G, AIC~8878).
